# A functional assay for the clinical annotation of genetic variants of uncertain significance in Diamond–Blackfan anemia

**DOI:** 10.1002/humu.23551

**Published:** 2018-05-28

**Authors:** Anna Aspesi, Marta Betti, Marika Sculco, Chiara Actis, Cristina Olgasi, Marcin W. Wlodarski, Adrianna Vlachos, Jeffrey M. Lipton, Ugo Ramenghi, Claudio Santoro, Antonia Follenzi, Steven R. Ellis, Irma Dianzani

**Affiliations:** ^1^ Department of Health Sciences Università del Piemonte Orientale Novara Italy; ^2^ Department of Pediatrics and Adolescent Medicine, Division of Pediatric Hematology and Oncology, Medical Center, Faculty of Medicine University of Freiburg Freiburg Germany; ^3^ Feinstein Institute for Medical Research Manhasset New York; ^4^ Division of Hematology/Oncology and Stem Cell Transplantation Cohen Children's Medical Center of New York New Hyde Park New York; ^5^ Department of Public Health and Pediatric Sciences University of Torino Torino Italy; ^6^ Department of Biochemistry and Molecular Genetics University of Louisville Louisville Kentucky

**Keywords:** Diamond–Blackfan anemia, functional assay, ribosomal protein, RPS19, VUS

## Abstract

Diamond–Blackfan anemia (DBA) is a rare genetic hypoplasia of erythroid progenitors characterized by mild to severe anemia and associated with congenital malformations. Clinical manifestations in DBA patients are quite variable and genetic testing has become a critical factor in establishing a diagnosis of DBA. The majority of DBA cases are due to heterozygous loss‐of‐function mutations in ribosomal protein (RP) genes. Causative mutations are fairly straightforward to identify in the case of large deletions and frameshift and nonsense mutations found early in a protein coding sequence, but diagnosis becomes more challenging in the case of missense mutations and small in‐frame indels. Our group recently characterized the phenotype of lymphoblastoid cell lines established from DBA patients with pathogenic lesions in *RPS19* and observed that defective pre‐rRNA processing, a hallmark of the disease, was rescued by lentiviral vectors expressing wild‐type RPS19. Here, we use this complementation assay to determine whether RPS19 variants of unknown significance are capable of rescuing pre‐rRNA processing defects in these lymphoblastoid cells as a means of assessing the effects of these sequence changes on the function of the RPS19 protein. This approach will be useful in differentiating pathogenic mutations from benign polymorphisms in identifying causative genes in DBA patients.

## 1 INTRODUCTION

Diamond–Blackfan anemia (DBA) is a congenital disorder of the bone marrow characterized by normochromic macrocytic anemia and associated with physical malformations and increased risk of malignancies (Lipton & Ellis, 2009; Vlachos et al., 2008). The penetrance is incomplete and a wide range of clinical manifestations may occur even among affected members of the same family. First‐line therapy is steroid treatment; options in steroid‐resistant patients are chronic transfusions or hematopoietic stem cell transplantation. DBA is usually caused by heterozygous mutations in ribosomal protein (RP) genes that lead to haploinsufficiency. To date, mutations in 19 RP genes (*RPS19, RPS24, RPS17, RPL35A, RPL5, RPL11, RPS7, RPS10, RPS26, RPL26, RPL15, RPL31, RPS29, RPS28, RPL27, RPS27, RPS15A, RPL35, RPL18*) have been identified in DBA patients (Cmejla, Cmejlova, Handrkova, Petrak, & Pospisilova, 2007; Doherty et al., 2010; Draptchinskaia et al., 1999; Farrar et al., 2008, 2014; Gazda et al., 2006, 2008, 2012; Gripp et al., 2014; Ikeda et al., 2017; Landowski et al., 2013; Mirabello et al., 2014, 2017; Wang et al., 2015). Rare mutations in *GATA1*, that abrogate the production of the full‐length protein (Parrella et al., 2014; Sankaran et al., 2012), and in *TSR2*, encoding a RPS26 interactor (Gripp et al., 2014), have also been described.

In DBA, the deficiency of a RP leads to the reduction in the number of ribosomes and this is particularly harmful for the red cell progenitors. Ribosome biogenesis is a complex process that requires the involvement of hundreds of different structural and accessory molecules. Mature 18S ribosomal RNA (rRNA), which forms the 40S subunit, together with mature 28S and 5.8S rRNAs, which are components of the 60S subunit, are all produced by sequential nucleolytic cleavages of a large polycistronic 45S precursor. Mutations in RPs of the small (RPS) or large (RPL) ribosomal subunit affect various steps of pre‐rRNA maturation, resulting in the impairment of ribosome biogenesis and function. Since the alterations of pre‐rRNA processing cause the accumulation of specific rRNA precursors depending on the mutated RP gene (Boria et al., 2010; Farrar et al., 2014; Flygare et al., 2007), pre‐rRNA analysis has been proposed as a potential aid for making a DBA diagnosis (Farrar et al., 2014; Quarello et al., 2016). RPS19 haploinsufficiency affects the maturation of 40S ribosomal subunits by specifically affecting the conversion of 21S pre‐rRNA into 18SE pre‐rRNA (Choesmel et al., 2007; Flygare et al., 2007), thus leading to accumulation of 21S pre‐rRNA and increase of 21S/18SE ratio.

While many of the mutations found in RP genes are nonsense, frameshift, and splice site mutations that can be easily interpreted as pathogenic based on their predicted effects on the expression of a protein, the pathogenicity of missense variants remains often controversial. Over 25% of all DBA patients’ mutations are found in *RPS19* and more than 40 different missense mutations and small in‐frame indels have been described in this gene (Boria et al., 2010; Konno et al., 2010; Ozono et al., 2016; Smetanina et al., 2015; Wang et al., 2015). Their pathogenic significance is often difficult to evaluate and may present an obstacle to genetic testing of the proband as well as of family members who are silent carriers of the disease. This is particularly relevant when trying to identify a suitable donor for hematopoietic stem cell transplantation. To be able to counsel patients in these families, it is necessary to fully understand the role played by these variants of unknown significance (VUS) on protein expression and function.

*In silico* tools can aid in the interpretation of VUS and rely on the following criteria: (i) absence or very low frequency of the variant in the general population, (ii) change of an evolutionary conserved codon, (iii) non‐conservative amino acid substitution, (iv) cosegregation of the variant with the disease phenotype in the family under study (Richards et al., 2015). However, it is not recommended to use these predictions as the sole source of evidence to reach a diagnostic conclusion, and functional studies should support the *in silico* results. A VUS in *RPS19* can be classified as benign when the analysis of rRNA maturation in patient cells points to defects in a different gene. On the contrary, the observation of pre‐rRNA processing alterations consistent with RPS19 loss of function, is not sufficient to interpret the VUS as pathogenic, since the patient could simultaneously carry a disease‐causing mutation, responsible for the defective rRNA processing, in another RP gene.

**Figure 1:**
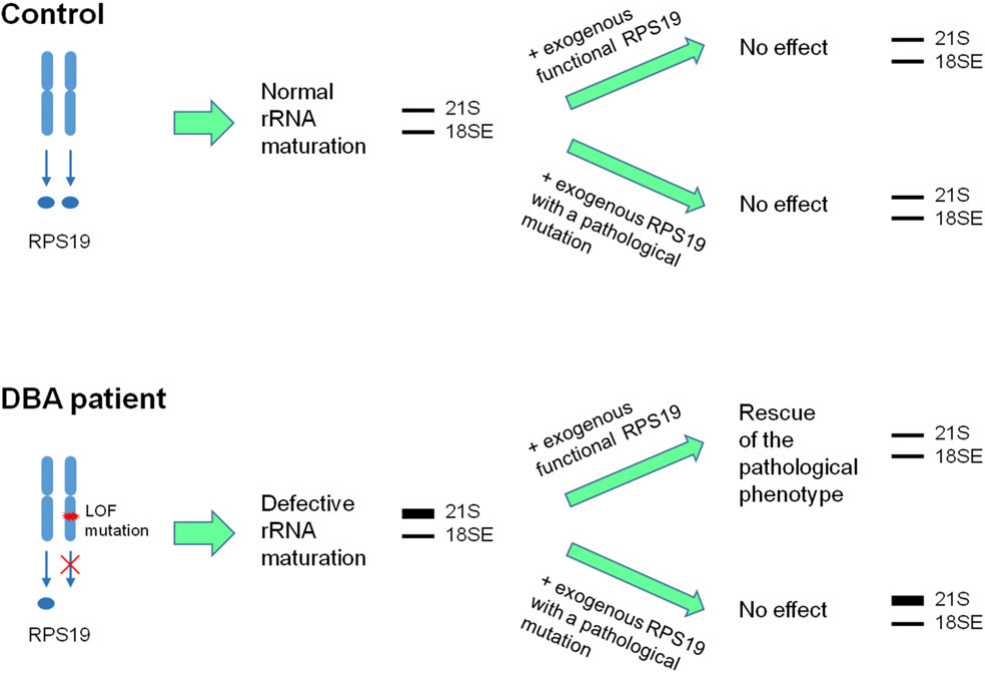
Scheme of the complementation assay. Cells from DBA patients have a loss‐of‐function mutation in *RPS19* that cause the accumulation of 21S rRNA. Expression of either wild‐type RPS19 or RPS19 with a benign sequence variant allows the rescue of the rRNA processing defect. On the contrary, expression of a RPS19 transgene carrying a deleterious mutation does not recover the pathological phenotype

We recently characterized the phenotype of lymphoblastoid cell lines (LCLs) established from DBA patients with loss‐of‐function mutations in *RPS19* (Aspesi et al., 2017). Aberrant pre‐rRNA processing and other pathological features were rescued by gene complementation, using an RPS19 transgene carried by a lentiviral vector (Aspesi et al., 2017). We reasoned that this complementation assay could be employed to investigate the effects of a wide range of VUS on RPS19 function (Figure 1). Here, we reviewed the literature regarding *RPS19* mutations and selected a total of 12 *RPS19* variants for functional analysis.

## 2 MATERIALS AND METHODS

### 2.1 Cells

LCLs were established as described previously from two DBA patients (P1 and P2) and one healthy subject (C), after approval of the local ethics committee (Aspesi et al., 2017). Both patients had a heterozygous loss‐of‐function mutation in *RPS19* and their genotypes were: P1: c.283_284delG, p.Gly95Alafs*16; P2: c.36_37insAG, p.Glu13Argfs*17 (RefSeq NG_007080.3, NM_001022.3, NP_001013.1). Cells were maintained in RPMI 1640 medium supplemented with 10% fetal bovine serum and antibiotics (100 U/ml penicillin and 100 μg/ml streptomycin) and were incubated at 37°C in a humidified atmosphere with 5% CO_2_.

### 2.2 Selection of variants for the complementation assay

From the list of 165 *RPS19* mutations described in the literature, we excluded protein‐truncating variants, that is, variants predicted to lead to nonsense‐mediated decay (NMD) and expected to have severe effects on gene function, as well as variants that disrupt a canonical splice site. For the present analysis, we considered only VUS: missense variants, small in‐frame indels, and truncating variants located in the last or penultimate exon. Stop codons located in the penultimate exon less than 50–55 bases from the final intron are not supposed to cause NMD (Le Hir, Izaurralde, Maquat, & Moore, 2000), thus the three nonsense variants in our list (c.376C>T p.Gln126*, c.382C>T p.Gln128*, c.406G>T p.Gly136*) could theoretically produce truncated proteins. We chose to select for the complementation assay only the variant located closest to the 3′ end of the transcript, c.406G>T p.Gly136*.

The novel variant c.338_340delTGG p.Val113del was submitted to the DBA database LOVD v.2.0 Build 36 (Boria et al., 2008, 2010).

To predict the functional consequences of variants, we used the following *in silico* prediction tools: SIFT v.1.03, Polyphen‐2 (Polymorphism Phenotyping v2), Provean v1.1.3, Condel 2.0, Mutation Assessor release 3, and MutationTaster 2. Mutation Assessor evaluates the probability that a mutation affects protein function, therefore the output “low” indicates a neutral variant. For Condel, the score ranges from 0 (neutral) to 1 (damaging); all variants we studied had an output between 0.5 and 0.9 and we considered them “probably damaging.” To address the potential effects on gene splicing, we used GeneSplicer, Human Splicing Finder version 3.1, MaxEntScan, NetGene2 version 2.42, NNSplice version 0.9, and FSPLICE. The presence and frequency of VUS selected for the complementation assay were evaluated in population databases: 1000 Genomes, Genome Aggregation Database (GnomAD), Exome Variant Server (EVS), and Exome Aggregation Consortium (ExAC).

### 2.3 Websites

DBA database, https://www.dbagenes.unito.it/home.php
SIFT, https://sift.jcvi.org
Polyphen‐2, https://genetics.bwh.harvard.edu/pph2
Provean, https://provean.jcvi.org/index.php
Condel, https://bbglab.irbbarcelona.org/fannsdb/
Mutation Assessor, https://mutationassessor.org
MutationTaster, https://www.mutationtaster.org
GeneSplicer, https://www.cbcb.umd.edu/software/GeneSplicer/gene_spl.shtml
Human Splicing Finder, https://www.umd.be/HSF
MaxEntScan, https://genes.mit.edu/burgelab/maxent/Xmaxentscan_scoreseq.html
NetGene2, https://www.cbs.dtu.dk/services/NetGene2
NNSplice. https://www.fruitfly.org/seq_tools/splice.html
FSPLICE, https://www.softberry.com/berry.phtml?topic=fsplice&group=programs&subgroup=gfind
1000 Genomes, https://www.internationalgenome.org/
GnomAD, https://gnomad.broadinstitute.org/
EVS, https://evs.gs.washington.edu/EVS/
ExAC, https://exac.broadinstitute.org/

### 2.4 Complementation assay

**Figure 2:**
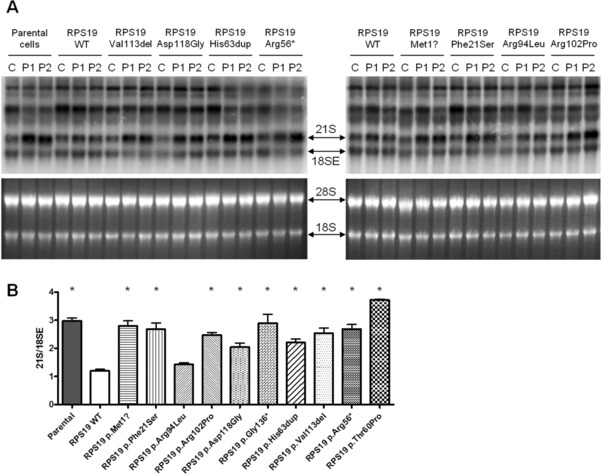
Complementation assay on VUS reported in DBA patients. **A**: Representative Northern blot experiments. Patient cells have an increased 21S/18SE rRNA ratio, that is corrected by the expression of a wild‐type RPS19 transgene but not by the expression of RPS19 carrying a pathogenic mutation. Upper panels show Northern blotting, lower panels show corresponding RNA gels stained by a fluorescent nucleic acid dye. C: control, P1: patient 1, P2: patient 2. **B**: Densitometry quantification of 21S/18SE ratio calculated on repeated Northern blot experiments. Asterisks represent statistically significant differences (*P* < 0.05) between samples with wild‐type and mutant exogenous RPS19. Error bars represent standard error of the mean

Site‐directed mutagenesis on RPS19 cDNA was carried out to introduce the selected variants using the QuikChange Site directed Mutagenesis kit (Agilent Technologies, Santa Clara, CA, USA). Primers are available upon request. The presence of each mutation was confirmed by Sanger sequencing. Lentiviral vectors were produced after transient transfection of 293T cells with the third generation packaging plasmids (pMDLg/pRRE, pRSV‐REV, and pMD2‐VSVG) and with the transfer construct for each RPS19 transgene (Aspesi et al., 2014; Follenzi, Ailles, Bakovic, Geuna, & Naldini, 2000). One control (C) and two RPS19‐haploinsufficient LCLs (P1 and P2) were transduced with 10 multiplicity of infection to express the mutant RPs. Integration and expression of at least one copy of the cassette carrying both RPS19 and green fluorescence protein (GFP) sequences resulted in the emission of green fluorescence. GFP^+^ cells were sorted, recultured for 2–3 weeks and analyzed. Total RNA was isolated using TRIzol Reagent (Invitrogen, Carlsbad, CA, USA), followed by on‐column DNase treatment and purification with miRNeasy Mini Kit (Qiagen, Milano, Italy). For Northern blot analysis, 5 μg of total RNA was fractionated on 1.5% formaldehyde agarose gels, transferred to a positively charged nylon membrane (Roche, Monza, Italy) and immobilized on the membrane by UV‐crosslinking performed with 120 milliJoules/cm^2^. The oligonucleotide probe (5′‐CCTCGCCCTCCGGGCTCCGTTAATGATC‐3′) was labeled with [γ‐^32^P]ATP using T4 polynucleotide kinase and hybridized overnight with the membrane at 37°C in ULTRAHyb‐Oligonucleotide hybridization buffer (Ambion, Thermo Fisher Scientific, Waltham, MA, USA). The membrane was washed at 37°C with 6XSSC and subjected to phosphorimaging analysis (Flygare et al., 2007). The overexpression of RPS19 did not cause adverse effects on pre‐rRNA processing in control cells (Aspesi et al., 2017), nor did the expression of RPS19 mutants (Figure 2A).

### 2.5 Quantitative RT‐PCR

RNA isolated from cells with wild‐type or mutant exogenous RPS19 was reverse transcribed using the High Capacity cDNA Reverse Transcription kit (Applied Biosystems, Foster City, CA, USA). Real‐time PCR amplification of cDNA was performed in triplicate using Power SYBR® Green PCR Master Mix (Applied Biosystems) and specific primers for the target genes *RPS19* and *CDKN1A* (p21). *ACTB* (β‐actin) was used as reference gene.

### 2.6 Statistical analysis

Northern blot bands were quantified using the ImageJ software. Results from P1 and P2 patient cells were considered as biological replicates. Differences in mean values between samples with either wild‐type or mutant exogenous RPS19 were analyzed with the Mann–Whitney test for two‐tailed data. Statistical significance was defined by a *P* value ≤0.05.

## 3 RESULTS

### 3.1 Selection of variants and *in silico* analyses

The DBA database (Boria et al., 2008, 2010) included 129 *RPS19* mutations in 219 patients at its last update in 2010. We reviewed the literature to collect additional *RPS19* mutations identified more recently and now have a total of 165 different *RPS19* mutations reported in 313 DBA patients (Arbiv et al., 2017; Chae et al., 2010, 2014; Da Costa et al., 2013; Delaporta et al., 2014; Errichiello et al., 2017; Farrar et al., 2011; Gerrard et al., 2013; Ichimura et al., 2017; Konno et al., 2010; Kuramitsu et al., 2012; Landowski et al., 2013; Ozono et al., 2016; Pospisilova et al., 2012; Quarello et al., 2012; Smetanina et al., 2015; Solomon et al., 2014; Tsangaris et al., 2011; van Dooijeweert et al., 2017; Wang et al., 2015; Zhang et al., 2016). For our study, we selected only those variants for which there was no strong evidence of pathogenicity according to the genetic criteria outlined in Materials and Methods, and obtained 47 VUS reported in 122 patients (39% of RPS19‐mutated patients, approximately 10% of all DBA patients). We also included a new previously unpublished variant we identified in a DBA patient by next‐generation sequencing, c.338_340delTGG p.Val113del (Supp. Table S1). Thirteen of the VUS have already been found to be pathogenic by published functional studies (Angelini et al., 2007; Badhai et al., 2009; Chatr‐Aryamontri et al., 2004; Chae et al., 2014; Choesmel et al., 2007; Cmejlova et al., 2006; Da Costa et al., 2003; Gazda et al., 2004; Hamaguchi et al., 2002; Idol et al., 2007;) (Supp. Table S1) and so were not tested in our study.

The VUS were analyzed by multiple *in silico* tools to predict the impact of sequence variants on protein function. Five missense variants that were analyzed were considered tolerated/benign by at least one of the six *in silico* tools indicating some degree of ambiguity as to whether or not they could be the pathogenic lesion in these patients. These were all selected for functional analysis using the complementation assay outlined here (Table 1). We also chose to study a nonsense VUS late in the coding sequence predicted to escape nonsense‐mediated mRNA decay (Le Hir et al., 2000) as well as two small in‐frame indels, whose impact could not be determined by most prediction algorithms (Table 1). As negative controls for the effect of known loss‐of‐function mutations, we chose a nonsense mutation, c.166C>T p.Arg56*, previously reported in four DBA patients (Boria et al., 2010; Proust et al., 2003; Willig et al., 1999) and the missense c.178A>C p.Thr60Pro, reported in one patient and predicted as damaging by every *in silico* tool we used. None of the variants chosen for functional analysis here were reported in 1000 Genomes, GnomAD, EVS, and ExAC.

**Table 1:** VUS in *RPS19* identified in DBA patients selected for complementation assay

DNA change	Protein change	Mutation taster	Mutation assessor	Polyphen‐2	Provean	SIFT	Condel	Affected patients
c.1A>G	p.Met1?	Disease causing	na	Probably damaging	Neutral	Damaging	Probably damaging	5
c.62T>C	p.Phe21Ser	Disease causing	Medium	Probably damaging	Deleterious	Tolerated	Probably damaging	1
c.187_189insCAC	p.His63dup	Disease causing	na	na	Deleterious	na	na	1
c.281G>T	p.Arg94Leu	Polymorphism	Medium	Benign	Deleterious	Tolerated	Probably damaging	1
c.305G>C	p.Arg102Pro	Disease causing	Medium	Benign	Deleterious	Damaging	Probably damaging	1
c.353A>G	p.Asp118Gly	Disease causing	Low	Benign	Deleterious	Tolerated	Probably damaging	1
c.406G>T	p.Gly136*	Disease causing	na	na	na	na	na	1
c.338_340delTGG	p.Val113del	Disease causing	na	na	Deleterious	na	na	1

na, not available. RefSeq: NM_001022.3, NP_001013.1.

Numeric output data are shown in Supp. Table S1. Websites and software versions are reported in the *Materials and Methods* section.

Finally, we assessed whether the VUS we selected caused the creation or loss of splice sites by *in silico* tools. These prediction tools have low specificity (∼60%–80%), but quite high sensitivity (∼90%–100%) in predicting splice site abnormalities, and therefore have a low false negative rate (Houdayer et al., 2012; Richards et al., 2015). None of the VUS included in this study were predicted to affect splicing, but the variant c.353A>G that, according to Human Splicing Finder, MaxEntScan, and FSPLICE, could activate a cryptic donor splice site (data not shown).

### 3.2 Analysis of VUS by complementation assay

Our previous findings showed that transfection of RPS19‐haploinsufficient LCLs with a vector expressing RPS19 cDNA corrected the abnormal accumulation of 21S pre‐rRNA, a well‐established defect in ribosome biogenesis caused by the loss of RPS19 function. Ribosome biogenesis defects such as these have been a hallmark of DBA pathogenesis in patients harboring mutations in RP genes. To assess the function of individual variants selected, we asked whether the variants could rescue the pre‐rRNA processing defect in the complementation assay outlined in Figure 1. Cells expressing the mutant transgenes showed an increased level of RPS19 transcript compared to parental cells, as measured by quantitative RT‐PCR (Supp. Figure S1A); Sanger sequencing performed on cDNA demonstrated the presence of the mutated transcripts (Supp. Figure S1B). Processing of pre‐rRNAs was evaluated by Northern blotting and results obtained by expressing wild‐type or mutant transgenes were compared. Representative experiments are shown in Figure 2A. Patient cells with no exogenous RPS19 (i.e., parental cells) or with the negative control p.Arg56* RPS19 had a mean 21S/18SE rRNA ratio of 2.97 ± 0.21 (standard deviation, SD) and 2.69 ± 0.31, respectively, whereas patient cells expressing the wild‐type transgene had a ratio of 1.20 ± 0.11, similar to the value of cells from healthy donors, that was 1.13 ± 0.13 (Figure 2A and B). Densitometry of Northern blots (Figure 2B) showed statistically significant differences (*P* < 0.05) between samples with wild‐type and mutant exogenous RPS19, demonstrating that all tested VUS were unable to recover the pathogenic phenotype of RPS19‐deficient cells. The only exception was c.281G>T p.Arg94Leu (mean 21S/18SE ratio 1.43 ± 0.12), which showed no statistical difference from wild‐type RPS19.

According to our data, we propose an arbitrary 21S/18SE cut‐off value ≥2 to define pathogenicity and ≤1.5 to indicate normal protein function. None of the mutants we analyzed showed a 21S/18SE ratio between these two values.

### 3.3 Analysis of variants found in population databases

**Figure 3:**
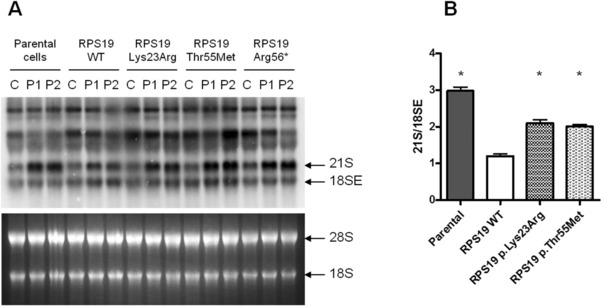
Complementation assay on VUS found in population databases. **A**: Representative Northern blot experiment. Upper panel shows Northern blot, lower panel shows the corresponding RNA gel stained by a fluorescent nucleic acid dye. C: control, P1: patient 1, P2: patient 2. **B**: Densitometry quantification of 21S/18SE ratio shows that neither variant could rescue the defective rRNA processing in patients cells. Asterisks represent statistically significant differences (*P* < 0.05) between samples with wild‐type and mutant exogenous RPS19. Error bars represent standard error of the mean

Population databases such as 1000 Genomes, GnomAD, EVS, and ExAC were searched for polymorphisms in *RPS19* that cause missense substitutions. Overall amino acid changes in RPS19 were extremely rare in population databases. No missense variant was present in 1000 Genomes, which includes data from more than 5,000 healthy subjects. The two most common variants in GnomAD, EVS, and ExAC, were c.68A>G p.Lys23Arg and c.164C>T p.Thr55Met (Table 2). These variants are presumed to be benign since it seems unlikely that individuals included in these databases would have a rare disease like DBA. Interestingly, variants c.68A>G p.Lys23Arg and c.164C>T p.Thr55Met were predicted to be benign only by one out of six and two out of six bioinformatic tools, respectively. Northern blot analysis showed that these variants failed to rescue the pre‐rRNA processing defects in patient lymphoblasts (Figure 3A). The mean 21S/18SE ratios for variants p.Lys23Arg and p.Thr55Met were 2.09 ± 0.13 and 2.01 ± 0.07, respectively, and were significantly different from data obtained by the wild‐type transgene, suggesting that these amino acid substitutions impair, at least partially, protein function (Figure 3A and B).

**Table 2:** VUS in *RPS19* selected from population databases

		Population database (MAF)									
DNA change	Protein change	1000 genomes	GnomAD	ExAC	EVS	Mutation taster	Mutation assessor	Polyphen‐2	Provean	SIFT	Condel
c.68A>G	p.Lys23Arg	Not reported	0.0001292	0.0001895	0.0002071	Disease causing score 26	Medium score 2.29	Benign score 0.097	Deleterious score −2.601	Damaging score 0.03	Probably damaging score 0.617
c.164C>T	p.Thr55Met	Not reported	0.0002261	0.0002734	0.0002478	Disease causing score 81	Medium score 2.08	Possibly damaging score 0.926	Neutral score −1.889	Tolerated score 0.06	Probably damaging score 0.647

MAF, minor allele frequency. RefSeq: NM_001022.3, NP_001013.1.

Mutation Taster: the score for amino acid substitutions reflects the physicochemical difference between the original and the mutated amino acid but does not influence the prediction. Mutation Assessor: the Functional Impact score is reported. PROVEAN: a score equal to or below the predefined threshold (−2.5) predicts a deleterious effect for the protein variant; a score above the threshold indicates that the variant is predicted to have a neutral effect. SIFT: the score predicts whether an amino acid substitution affects protein function, and ranges from 0.0 (deleterious) to 1.0 (tolerated).

Websites and software versions are shown in the *Materials and Methods* section.

### 3.4 Evaluation of p21 transcript level

**Figure 4:**
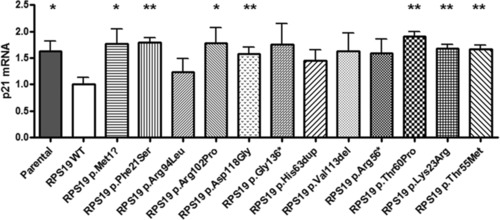
Level of p21 transcript in patient cells expressing wild‐type or mutant RPS19. The increased level of p21 typical of DBA patient cells is corrected by the expression of a wild‐type RPS19 transgene but not by the expression of RPS19 carrying a pathogenic mutation. Results obtained by quantitative RT‐PCR performed on P1 and P2 patient cells were considered biological replicates. Asterisks represent statistically significant differences between samples with wild‐type and mutant exogenous RPS19: *P < 0.05; **P < 0.01. Error bars represent standard error of the mean

RPS19 deficiency induces stabilization of p53 and increased level of its target p21; such alterations are recovered by expression of the RPS19 transgene (Aspesi et al., 2017). We performed quantitative RT‐PCR to measure the level of p21 transcript in patient cells expressing the mutant transgenes. The results validated the data obtained by Northern blot analysis, since the expression of RPS19 mutants could not normalize the level of p21, but the expression of mutant p.Arg94Leu led to a clear, though not significant, decrease of p21 (Figure 4). Interestingly, the high p21 levels measured for the VUS p.Lys23Arg and p.Thr55Met corroborate their interpretation as deleterious variants (Figure 4).

## 4 DISCUSSION

Our work was aimed at creating a complementation assay to assess the function of RPS19 VUS and clarify their involvement in the pathogenesis of DBA. Novel sequence variants in genes known to be involved in DBA have been increasingly detected, but our understanding of the clinical significance of these novel variants is often limited. Many of the most challenging variants change only one amino acid, making it difficult to discern benign variants from pathogenic mutations that disrupt protein function. *In silico* prediction tools can be of aid in the interpretation of sequence variants but their output should be validated by functional studies.

It is widely accepted that the underlying pathophysiology in the majority of DBA patients is haploinsufficiency for a RPs and an ensuing disruption of ribosome biogenesis. *RPS19* is the most frequently mutated gene in DBA and structure/function relationships of known pathogenic mutations have been characterized previously. The crystal structure of RPS19 from the archeon *Pyroccocus abyssi* was used to group RPS19 missense mutations in two classes: class I included residues essential for the folding and stability of the protein, whereas class II included mutations that affected surface residues and presumably impaired the capacity of RPS19 to engage intermolecular interactions (Gregory et al., 2007). In another study, eleven missense mutations and one trinucleotide insertion were expressed in human HEK293 cells (Angelini et al., 2007). Some mutants, corresponding to class I mutants (p.Val15Phe, p.Leu18Pro, p.Ala57Pro, p.Ala61Glu, p.Gly127Glu, and p.Leu18_Ala19insGlu) were expressed at a barely detectable level, whereas the others (all class II mutants: p.Pro47Leu, p.Trp52Arg, p.Arg56Gln, p.Arg62Gln, p.Arg62Trp, p.Arg101His), although more stable, were not assembled into mature ribosomes.

Additional studies have shown that missense mutations can influence the localization of RPS19 and disrupt function by mislocalization. For example, mutant proteins such as p.Val15Phe, p.Gly127Gln, and p.Leu131Pro could not reach the nucleolus and their expression was dramatically decreased compared with the wild‐type protein (Cretien et al., 2008; Da Costa et al., 2003).

Because of the limitations associated with assessing a protein's function based on sequence analysis alone, there is a significant need to be able to functionally annotate novel sequence variants for their potential involvement in DBA pathogenesis. We have developed a new complementation assay to assess a variant's function based on its ability to rescue a pre‐rRNA processing defect in lymphoblasts derived from patients haploinsufficient for RPS19. This assay is not restricted to RPS19 and could potentially be developed for other known DBA genes.

We functionally tested 12 variants: nine were already described in the literature as mutations responsible for DBA, one was a new variant we identified in a DBA patient and two variants were selected from population databases. Among the variants we analyzed, only the missense c.281G>T p.Arg94Leu successfully rescued the rRNA processing defect in patient cells. This variant was reported as pathogenic after the identification in a female DBA patient whose parents were not tested (Boria et al., 2010). Our results suggest that this variant may actually be benign and further studies should be carried out in this patient to establish the causative gene. The remaining variants c.1A>G p.Met1?, c.62T>C p.Phe21Ser, c.187_189insCAC p.His63dup, c.305G>C p.Arg102Pro, c.353A>G p.Asp118Gly and c.406G>T p.Gly136* were not able to complement RPS19 deficiency. Among the variant failing to complement was the new variant c.338_340delTGG p.Val113del, whose impact was difficult to predict even by bioinformatic algorithms.

The most surprising result from our analysis was that two rare variants selected from population databases, c.68A>G p.Lys23Arg, and c.164C>T p.Thr55Met failed to complement the pre‐rRNA processing defect in patient lymphoblasts. The latter variant was previously observed in a DBA patient who also carried a second variant, p.Val15Phe, on the same allele. In this case, p.Val15Phe was considered pathogenic, whereas p.Thr55Met was interpreted as benign because only protein localization was being studied (Boria et al., 2008; Da Costa et al., 2003).

There is growing evidence that RP mutations can be found in patients with very mild or absent hematologic manifestations, as previously described, for instance, in a family with no sign of DBA where a truncating germline mutation in *RPS20* cosegregated with colon cancer (Nieminen et al., 2014). This is also supported by the recent report of two unrelated patients with congenital heart disease and mutations in *RPS24* who were not anemic (Vlachos et al., 2018). Our observation that VUS reported in population databases could be involved in DBA pathogenesis highlights the need to deepen our knowledge about the possible presence in the general population of silent carriers of RP mutations and atypical cases of DBA.

Our results also emphasize the limited reliability of *in silico* tools for pathogenicity prediction. According to the data obtained by our complementation assay, Mutation Taster was the only tool with sensitivity and specificity equal to 1, whereas the other tools resulted in false negative and/or false positive predictions.

Several VUS have been reported also in other *RP* genes mutated in DBA patients (Arbiv et al., 2017; Doherty et al., 2010; Gerrard et al., 2013; Konno et al., 2010; Pospisilova et al., 2012; Smetanina et al., 2015; Tsangaris et al., 2011; van Dooijeweert et al., 2017). In the future, appropriate complementation assays could be implemented to extend the study of pathogenicity to other DBA genes, similarly to the approach we developed for *RPS19*.

In conclusion, we provided a strategy to distinguish disease‐causing mutations in *RPS19* from benign polymorphisms and clarify their clinical significance. This information should assist clinicians in the counseling and management of DBA patients and their families.

## Supporting information

SUPPLEMENTARY MATERIAL
