# Supplementary material for: A functional assay for the clinical annotation of genetic variants of uncertain significance in Diamond–Blackfan anemia
Source: Hum Mutat. 2018 May 28;39(8):1102–11. doi: 10.1002/humu.23551 (PMC6055729; doi:10.1002/humu.23551)
Supplement: Supplementary file 1 — SUPPLEMENTARY MATERIAL [file HUMU-39-1102-s001.pdf]

SUPPLEMENTARY MATERIAL

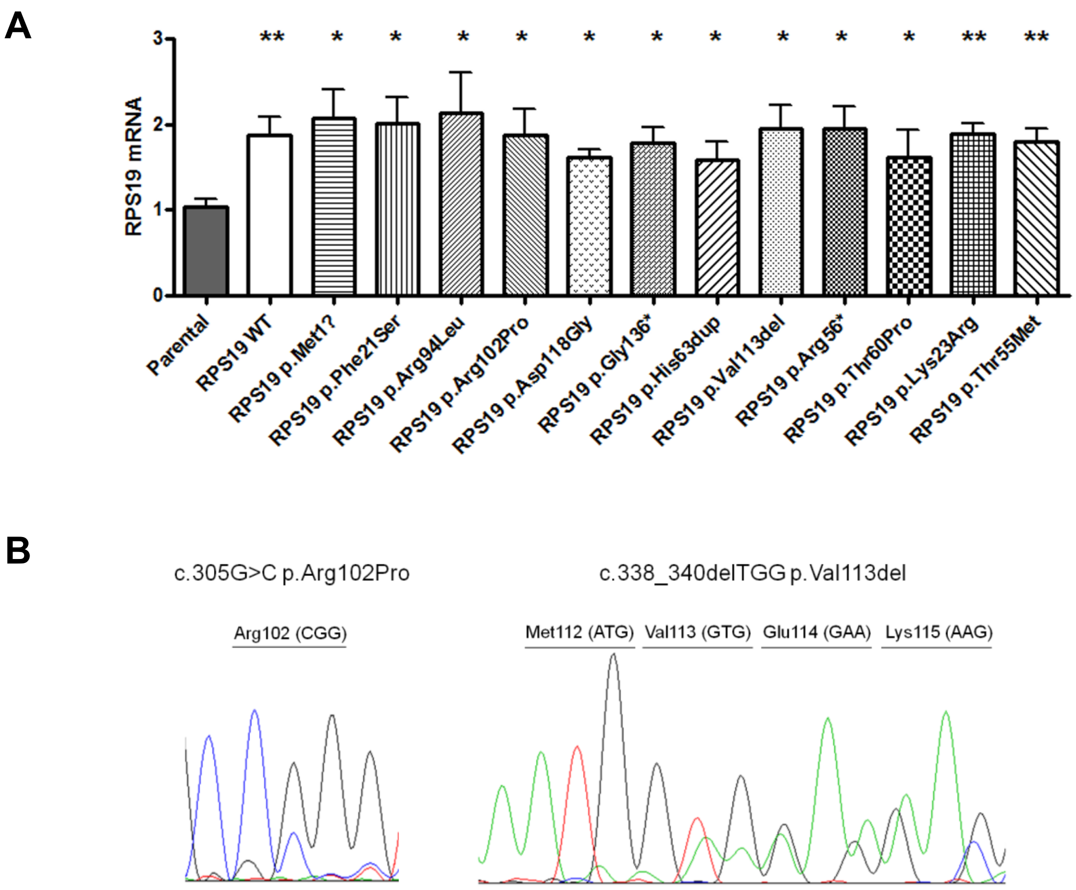

Supplementary Figure S1. *RPS19* transcript in patient cells expressing wild-type or mutant exogenous RPS19. (**A**) The level of *RPS19* transcript measured by quantitative RT-PCR was increased after transduction with RPS19 transgenes. Asterisks represent statistically significant differences between parental cells and cells expressing exogenous RPS19: * $p<0.05$; ** $p<0.01$. Results obtained from P1 and P2 patient cells were considered biological replicates. Error bars represent standard error of the mean. (**B**) Sanger sequencing performed on cDNA demonstrated the presence of each mutation in *RPS19* transcript. Representative electropherograms of cDNA obtained from cells expressing mutants c.305G>C p.Arg102Pro and c.338_340delTGG p.Val113del are shown.

Supplementary Table S1. VUS in *RPS19* identified in DBA patients.

ID	DNA change	Protein change	Mutation Taster	Mutation Assessor	Polyphen-2	PROVEAN	SIFT	Condel	Affected patients	Functional studies	Pathogenic according to functional studies
1	c.1A>G	p.Met1?	Disease causing M1? initiating Methionine lost	na	Probably damaging score 1	Neutral score -2.491	Damaging score 0	Probably damaging score 0.577	5	Chatr-Aryamontri et al (2004); Badhai et al (2009)	yes (this work)
2	c.2T>A	p.Met1?	Disease causing M1? initiating Methionine lost	na	Probably damaging score 1	Deleterious score -3.618	Damaging score 0	Probably damaging score 0.582	1	/	/
3	c.3G>A	p.Met1?	Disease causing M1? initiating Methionine lost	na	Probably damaging score 1	Deleterious score -2.526	Damaging score 0	Probably damaging score 0.577	9	Chae et al (2014)	yes
4	c.3G>C	p.Met1?	Disease causing M1? initiating Methionine lost	na	Probably damaging score 1	Deleterious score -2.526	Damaging score 0	Probably damaging score 0.577	1	/	/
5	c.3G>T	p.Met1?	Disease causing M1? initiating Methionine lost	na	Probably damaging score 1	Deleterious score -2.526	Damaging score 0	Probably damaging score 0.577	9	/	/
6	c.26T>A	p.Val9Glu	Disease causing score 121	High score 3.84	Probably damaging score 0.996	Deleterious score -6.395	Damaging score 0	Probably damaging score 0.828	1	/	/
7	c.43G>T	p.Val15Phe	Disease causing score 50	Medium score 3.395	Probably damaging score 0.997	Deleterious score -3.937	Damaging score 0	Probably damaging score 0.690	2	Angelini et al (2007); Da Costa et al (2003)	yes
8	c.49G>C	p.Ala17Pro	Disease causing score 27	Medium score 2.845	Probably damaging score 0.999	Deleterious score -4.048	Damaging score 0.01	Probably damaging score 0.719	3	/	/
9	c.53T>C	p.Leu18Pro	Disease causing score 98	Medium score 3.265	Probably damaging score 0.997	Deleterious score -5.270	Damaging score 0	Probably damaging score 0.772	1	Angelini et al (2007)	yes
10	c.53T>G	p.Leu18Arg	Disease causing score 102	Medium score 3.265	Probably damaging score 0.990	Deleterious score -4.619	Damaging score 0	Probably damaging score 0.772	1	/	/
11	c.53_54insAGA	p.Leu18_Ala19insGlu	Disease causing	na	na	Deleterious score -9.224	na	na	1	Angelini et al (2007)	yes

12	c.58G>C	p.Ala20Pro	Disease causing score 27	Medium score 3.43	Probably damaging score 0.996	Deleterious score -4.059	Damaging score 0.03	Probably damaging score 0.778	1	/	/
13	c.62T>C	p.Phe21Ser	Disease causing score 155	Medium score 2.44	Probably damaging score 0.969	Deleterious score -6.578	Tolerated score 0.07	Probably damaging score 0.780	1	/	yes (this work)
14	c.83T>G	p.Leu28Arg	Disease causing score 102	Medium score 3.365	Probably damaging score 0.983	Deleterious score -4.487	Damaging score 0	Probably damaging score 0.767	1	/	/
15	c.114G>C	p.Lys38Asn	Disease causing score 94	High score 3.93	Probably damaging score 0.999	Deleterious score -4.257	Damaging score 0	Probably damaging score 0.836	1	/	/
16	c.140C>T	p.Pro47Leu	Disease causing score 98	High score 3.89	Possibly damaging score 0.945	Deleterious score -8.823	Damaging score 0.01	Probably damaging score 0.832	2	Angelini et al (2007); Idol et al (2007)	yes
17	c.154T>C	p.Trp52Arg	Disease causing score 101	High score 3.925	Probably damaging score 0.999	Deleterious score -13.176	Damaging score 0	Probably damaging score 0.834	2	Angelini et al (2007)	yes
18	c.156G>C	p.Trp52Cys	Disease causing score 215	High score 3.925	Probably damaging score 1	Deleterious score -12.234	Damaging score 0	Probably damaging score 0.834	2	/	/
19	c.167G>A	p.Arg56Gln	Disease causing score 43	High score 3.835	Possibly damaging score 0.900	Deleterious score -3.468	Damaging score 0.01	Probably damaging score 0.749	9	Angelini et al (2007); Da Costa et al (2003); Cmejlova et al (2006)	yes
20	c.167G>C	p.Arg56Pro	Disease causing score 103	High score 3.835	Probably damaging score 1	Deleterious score -6.360	Damaging score 0.01	Probably damaging score 0.818	2	/	/
21	c.169G>C	p.Ala57Pro	Disease causing score 27	High score 3.54	Probably damaging score 0.985	Deleterious score -3.807	Damaging score 0	Probably damaging score 0.789	1	Angelini et al (2007)	yes
22	c.172G>C	p.Ala58Pro	Disease causing score 27	High score 3.89	Probably damaging score 1	Deleterious score -4.721	Damaging score 0	Probably damaging score 0.831	1	/	/
23	c.176C>T	p.Ser59Phe	Disease causing score 155	High score 3.695	Probably damaging score 1	Deleterious score -5.247	Damaging score 0	Probably damaging score 0.811	1	/	/
24	c.178A>C	p.Thr60Pro	Disease causing score 38	Medium score 2.555	Probably damaging score 0.984	Deleterious score -3.474	Damaging score 0	Probably damaging score 0.676	1	/	/

25	c.182C>A	p.Ala61Glu	Disease causing score 107	Medium score 3.35	Probably damaging score 0.969	Deleterious score -3.790	Damaging score 0.02	Probably damaging score 0.675	1	Angelini et al (2007)	yes
26	c.184C>T	p.Arg62Trp	Disease causing score 101	High score 3.89	Probably damaging score 0.987	Deleterious score -7.487	Damaging score 0.02	Probably damaging score 0.825	18	Angelini et al (2007); Da Costa et al (2003); Hamaguchi et al (2002)	yes
27	c.185G>A	p.Arg62Gln	Disease causing score 43	High score 3,89	Possibly damaging score 0.817	Deleterious score -3.762	Damaging score 0.01	Probably damaging score 0.723	17	Angelini et al (2007); Choesmel et al (2007)	yes
28	c.187_189insCAC	p.His63dup	Disease causing	na	na	Deleterious score -9.127	na	na	1	/	yes (this work)
29	c.191T>C	p.Leu64Pro	Disease causing score 98	Medium score 3.45	Probably damaging score 1	Deleterious score -6.137	Damaging score 0	Probably damaging score 0.788	1	/	/
30	c.212G>A	p.Gly71Glu	Disease causing score 98	High score 3.885	Probably damaging score 1	Deleterious score -7.264	Damaging score 0	Probably damaging score 0.824	1	/	/
31	c.226A>C	p.Thr76Pro	Disease causing score 38	Medium score 2.93	Possibly damaging score 0.728	Deleterious score -3.760	Damaging score 0.04	Probably damaging score 0.750	1	/	/
32	c.281G>T	p.Arg94Leu	Polymorphism score 102	Medium score 2.05	Benign score 0.006	Deleterious score -3.850	Tolerated score 0.25	Probably damaging score 0.610	1	/	no (this work)
33	c.284G>T	p.Gly95Val	Disease causing score 109	Medium score 2.95	Possibly damaging score 0.741	Deleterious score -5.142	Damaging score 0.02	Probably damaging score 0.781	1	/	/
34	c.301C>T	p.Arg101Cys	Disease causing score 180	Medium score 3.08	Possibly damaging score 0.888	Deleterious score -6.340	Damaging score 0.01	Probably damaging score 0.721	1	/	/
35	c.302G>A	p.Arg101His	Disease causing score 29	Medium score 2.455	Possibly damaging score 0.576	Deleterious score -4.091	Damaging score 0.04	Probably damaging score 0.673	7	Angelini et al (2007); Gazda et al (2004)	yes
36	c.305G>C	p.Arg102Pro	Disease causing score 103	Medium score 2.11	Benign score 0.001	Deleterious score -3.590	Damaging score 0.02	Probably damaging score 0.593	1	/	yes (this work)
37	c.320T>G	p.Leu107Arg	Disease causing score 102	Medium score 3.19	Probably damaging score 0.969	Deleterious score -5.882	Damaging score 0.01	Probably damaging score 0.837	1	/	/

38	c.353A>G	p.Asp118Gly	Disease causing score 94	Low score 1.29	Benign score 0.045	Deleterious score -2.577	Tolerated score 0.21	Probably damaging score 0.603	1	/	yes (this work)
39	c.358G>A	p.Gly120Ser	Disease causing score 56	High score 3.885	Probably damaging score 1.000	Deleterious score -5.867	Damaging score 0.02	Probably damaging score 0.796	1	/	/
40	c.376C>T	p.Gln126*	Disease causing	na	na	na	na	na	2	/	/
41	c.380G>A	p.Gly127Glu	Disease causing score 98	Medium score 2.73	Probably damaging score 0.988	Deleterious score -7.117	Damaging score 0.02	Probably damaging score 0.762	1	Angelini et al (2007); Da Costa et al (2003)	yes
42	c.382C>T	p.Gln128*	Disease causing	na	na	na	na	na	1	/	/
43	c.392T>C	p.Leu131Pro	Disease causing score 98	High score 3.77	Probably damaging score 1	Deleterious score -6.320	Damaging score 0	Probably damaging score 0.792	1	/	/
44	c.392T>G	p.Leu131Arg	Disease causing score 102	High score 3.77	Possibly damaging score 0.938	Deleterious score -5.429	Damaging score 0.01	Probably damaging score 0.826	2	/	/
45	c.403G>A	p.Ala135Thr	Disease causing score 58	High score 3.84	Possibly damaging score 0.949	Deleterious score -3.497	Damaging score 0.02	Probably damaging score 0.815	1	/	/
46	c.406G>T	p.Gly136*	Disease causing	na	na	na	na	na	1	/	yes (this work)
47	c.338_340delTGG	p.Val113del	Disease causing	na	na	Deleterious score -13.503	na	na	1	/	yes (this work)

na: not available. RefSeq: NM_001022.3, NP_001013.1.

Mutation Taster: the score for amino acid substitutions reflects the physicochemical difference between the original and the mutated amino acid but does not influence the prediction. Mutation Assessor: the Functional Impact score is reported. PROVEAN: a score equal to or below the predefined threshold (-2.5) predicts a deleterious effect for the protein variant; a score above the threshold indicates that the variant is predicted to have a neutral effect. SIFT: the score predicts whether an amino acid substitution affects protein function, and ranges from 0.0 (deleterious) to 1.0 (tolerated).

Websites and software versions are shown in the Method section.
